# Fortification of diets with omega-3 long-chain polyunsaturated fatty acids enhances feedlot performance, intramuscular fat content, fat melting point, and carcass characteristics of Tattykeel Australian White MARGRA lambs

**DOI:** 10.3389/fvets.2022.933038

**Published:** 2022-09-12

**Authors:** Shedrach Benjamin Pewan, John Roger Otto, Robert Tumwesigye Kinobe, Oyelola Abdulwasiu Adegboye, Aduli Enoch Othniel Malau-Aduli

**Affiliations:** ^1^Animal Genetics and Nutrition, Veterinary Sciences Discipline, College of Public Health, Medical and Veterinary Sciences, Division of Tropical Health and Medicine, James Cook University, Townsville, QLD, Australia; ^2^National Veterinary Research Institute, Vom, Plateau State, Nigeria; ^3^Public Health and Tropical Medicine Discipline, College of Public Health, Medical and Veterinary Sciences, Division of Tropical Health and Medicine, James Cook University, Townsville, QLD, Australia

**Keywords:** omega-3 fatty acids, meat eating quality, wholesale primal cuts, intramuscular fat, fat melting point, MARGRA lamb

## Abstract

Meat eating quality indices such as intramuscular fat content (IMF) and fat melting point (FMP) of the *Longissimus thoracis et lumborum* muscle and the feedlot performance, carcass traits, and commercial wholesale cuts of lot-fed Tattykeel Australian White (TAW) MARGRA lambs as a result of dietary fortification of the diet with omega-3 long-chain polyunsaturated fatty acids (n-3 LC-PUFA) were evaluated. A total of 75 TAW MARGRA lambs at 6 months of age with an average liveweight of 30 ± 1.2 kg were used. The lambs were randomly allocated to the following three dietary treatments of 25 lambs each in a 47-day feeding trial using a completely randomized experimental design: (1) control diet of hay plus pellets without omega-3 oil, (2) hay plus commercial whole grain pellets (MSM) without omega-3 oil, and (3) hay plus pellets fortified with omega-3 oil. It was hypothesized that dietary supplementation with omega-3 fortified pellets will improve feedlot performance, meat-eating quality indices of IMF, FMP, and carcass characteristics. Lot-fed lambs on the MSM whole grain had the highest feed intake of 1.69 kg/day, followed by the control at 1.57 kg/day and the lowest in the omega-3 diet at 1.01 kg/day (*p* = 0.0001). However, the omega-3 diet had the highest average daily gain of 230 g/head/day (*p* = 0.0001), indicating the greatest feed efficiency since it had the best growth response with minimal feed intake. Post-slaughter evaluation of the *Longissimus thoracis et lumborum* muscle revealed significant treatment variations in IMF (*p* = 0.0001), FMP (*p* = 0.0001), pH (*p* = 0.0380), and wholesale French rack primal cut (*p* = 0.0001). Strong correlations (*p* < 0.05) between liveweight, temperature, pH, FMP, and IMF were observed. Similarly, significant correlations between carcass characteristics of total saleable meat yield, lean trim, fat trims, bones, and leg shank were evident (*p* < 0.05). However, there were no treatment differences in the final liveweight, GR fat depth, hot standard carcass weight, or dressing percentage. The findings indicate that feedlot performance, meat-eating quality traits such as IMF and FMP, and commercial wholesale French rack cuts can be further improved during feedlot finishing of TAW MARGRA lambs through dietary supplementation with omega-3 oils, and hence the tested hypothesis of improved meat quality attributes is partially confirmed.

## Introduction

As the world's fourth most consumed meat after pork, poultry, and beef (1), lamb meat contributes significantly to global human nutrition since it contains nutrients of high biological value (2). In 2021, Australia was ranked second in global sheep production after China, and the latter still imports sheep meat as local production cannot meet its domestic needs (3). As the world's largest sheep exporter in 2021, Australia's overall sheep meat exports increased by 5.3% to $3.96 billion, representing a 0.5% increase in contribution to the overall worth of exports to the national economy (3).

Lamb consumers demand fresh, tasty, safe, and microbe-free meat with high eating quality and nutrient content, thus necessitating advanced nutrition and breeding strategies that integrate appropriate meat quality characteristics by sheep producers (4) to improve feedlot performance, dressing percentage, lean yield, and marbling score (5, 6). To achieve accelerated lamb growth and early attainment of appropriate slaughter weights that meet market specifications, feedlotting remains a critical lamb finishing strategy for improving profitability (7, 8). It also facilitates the production of more uniform lamb carcasses (9) from a low mortality system that ensures more efficient use of human and technical resources to attain improved meat yield and quality (10). However, increased feed cost is a major limiting factor in the feedlot system (11), accounting for 65%−70% of the total cost of small ruminant production (12). As such, nutritional strategies for increasing animal growth performance using cheap feeds without compromising carcass nutrient value and eating quality are essential elements for a profitable livestock production enterprise to consider (13). Hence, there is a need for concerted research efforts in exploring diverse dietary fortification options.

The red meat industry is experiencing modernization in its production system to meet current consumer demands associated with health, quality of life, and sustainability (14). The profile and quality of fatty acids in lamb can be improved by incorporating lipid sources into the diet of lot-fed lambs to boost the levels of omega-3 long-chain polyunsaturated fatty acids (n-3 LC PUFA) known to be beneficial for human health (14, 15). Meat industry producers and processors aim at producing heavy carcasses of young animals with good musculature, supplying desired meat cut yields and an attainable fat layer to protect carcasses while in cold storage (16). Intramuscular fat (IMF), fat melting point (FMP), and pH are essential quality indicators of red meat (17). The high marbling score represents the improved amount of IMF, water holding capacity, tenderness, flavor, juiciness, lamb palatability (18, 19), content, and distribution of protein in the muscle fibers (20).

To the best of our current knowledge of the published literature, this is the first study to fortify the diets of TAW MARGRA lambs with n-3 LC-PUFA to enhance feedlot performance, carcass characteristics, commercial wholesale cut yields, and meat eating quality traits.

## Materials and methods

### Animals, study location, dietary treatments, experimental design, and feed intake

The animals, study location, dietary treatments, and experimental design have already been described in detail by Pewan et al. (21). Briefly, TAW MARGRA lamb breed was developed from the rigorous selection, culling, and linebreeding of Texel, Van Rooy, Dorper, and Poll Dorset with an extensive utilization of natural mating, artificial insemination, and embryo transfer (21). TAW MARGRA lamb is a special breed of lamb with a low FMP (28–39^o^C) compared to the ranges of 40.6–48.0°C and 41.5–44.0°C reported in the study by Flakemore et al. (22) and Holman et al. (23) for purebred and crossbred Merino, Dorset, Black, and White Suffolk sheep. Similarly, TAW lambs contained EPA+DHA content of 32.4 ± 8.5 mg per 100 g of muscle, surpassing the 30 mg limit set by Food Standards of Australia and New Zealand (FSANZ) for the “source” claim (24).

The feedlot finishing feeding trial was performed at Crown Agriculture's lamb feedlot facility at Borenore, New South Wales, Australia, from April to June 2019. The study utilized 75 TAW MARGRA lambs at 6 months of age with an average liveweight of 30 ±1.2 kg. The lambs were dewormed and allowed a 14-day adjustment period with *ad libitum* access to water and the gradual introduction of three experimental diets to minimize any gastrointestinal disorders. The lambs were randomly allocated to the following three dietary treatments of 25 lambs each in a 47-day feeding trial using a completely randomized experimental design: (1) control diet of hay plus pellets without omega-3 oil, (2) hay plus commercial whole grain pellets (MSM) without omega-3 oil, and (3) hay plus pellets fortified with omega-3 oil. All lambs were fed in groups (control, MSM, and omega-3). All these three diets were formulated to be isonitrogenous (CP = 14%) and isocaloric (ME = 10.258 Mj/kg DM). Details of the nutrient compositions have been published by Pewan et al. (21). The feeding troughs had electronic sensors, where each animal's identification, liveweight, feed intake, average daily gains, and other vital parameters were automatically recorded, cloud-stored, and downloaded at the required time. At the end of the feeding trial, the lambs were transported to the Gundagai Meat Processing plant, held in lairage overnight, and humanely sacrificed as a single mob in line with Meat Standards Australia specifications. The carcasses were subjected to medium-voltage electrical stimulation before being trimmed and dressed (25). Carcasses were kept in the chiller room for 24 h during which *in situ* pH and temperature were recorded.

### Carcass measurements

Hot standard carcass weight was recorded prior to chilling, and cold carcass weight was measured after removing from the chiller at 4°C after 24 h. The dressing percentage was determined as the hot standard carcass weight (HSCW) divided by the liveweight (LWT) × 100%. In the boning room, the carcasses were cut between the 12th and 13th ribs to measure *longissimus dorsi* eye muscle area (LMA), back-fat thickness (BF), body wall thickness (BWT), loin marbling score, body and leg conformation scores, and the percentage of boneless, closely trimmed retail cuts (% BCTRC) as described previously by Jaborek et al. (26). Wholesale primal cuts, fat trims, lean, and bone weights were recorded.

### Determination of IMF

The technique of Flakemore et al. (27) was used to determine the IMF content of muscle samples. This was carried out by homogenizing and extracting in CHCl_3_:MeOH (2:1) fat-soluble solvent, phase partition in 5 ml of 10% KCl, and evaporation of the organic layer in weighed porcelain crucibles to get the fat content. The % IMF was computed as follows: [Crucible including fat weight (g) – empty crucible weight (g)]/sample weight (g) × 100.

### Determination of FMP

The FMP was determined as described by Mwangi et al. (28) and Pewan et al. (29). Briefly, the muscle samples were placed in an oven at 100°C for 1–2 min to obtain fat that was used for FMP determination. Through air suction, the melted fat was sucked into thin capillary tubes and kept in a refrigerator for 10 min at 4°C to permit the fat to freeze. The fat level was marked with an indelible pen, and the capillary tube was affixed to a thermometer held in a glass beaker with ~80 ml of deionised H_2_O positioned on a heating block. The heating block was slowly heated until the fat “slipped” off the mark. The temperature at which the “slip” occurred was recorded as the FMP.

### pH and temperature measurements

pH and temperature were recorded at the 12th to 13th rib from *longissimus lumborum* muscle from the left side of each carcass as documented by Holman et al. (25) and Hussain et al. (30). Briefly, the initial pH measurement was performed immediately upon entry into the chiller (~30 min post-slaughter). At ~24 h *post-mortem*, four intermediate measurements were taken before the last pH measurement. A pH meter (WP-80, TPS Pty Ltd., Queensland) fitted with a polypropylene spear-type gel electrode (IJ-44, Ionode™, TPS Pty Ltd., Queensland) and calibrated using pH 4.00 and pH 7.00 standards was used for all measurements. The pH meter was initially recalibrated at each interval using standard buffers at a temperature that matched the estimated muscle temperature. This was to compensate for the influence of temperature on pH readings, as per the technical bulletin (WP-80, TPS Pty Ltd., Queensland). Muscle temperature was concurrently documented with the aid of the same pH meter, tailored with a spear-type temperature sensor (no. 121247, TPS Pty Ltd., Queensland).

### Statistical analysis

The data were analyzed utilizing nonparametric analytical methods in R. Each animal was regarded as an experimental unit. Meat quality, carcass characteristics, and wholesale cut yields were presented as medians and interquartile range (IQR) and visualized in boxplots after adjustment for treatment effect. Spearman's correlation procedure was used to quantify the relationship between variables. To analyse the effect of treatment on FMP, IMF, and carcass traits, the treatment was defined as the fixed effect. The Kruskal–Wallis test was also used to decide whether or not a statistically significant difference existed between the different feed treatments as previously described (31). Tukey's adjusted multiple comparisons were also used for the pairwise comparison test at *p* < 0.05.

## Results

### Liveweight, average daily feed intake, average daily gain, and feed cost

**Figure 1:**
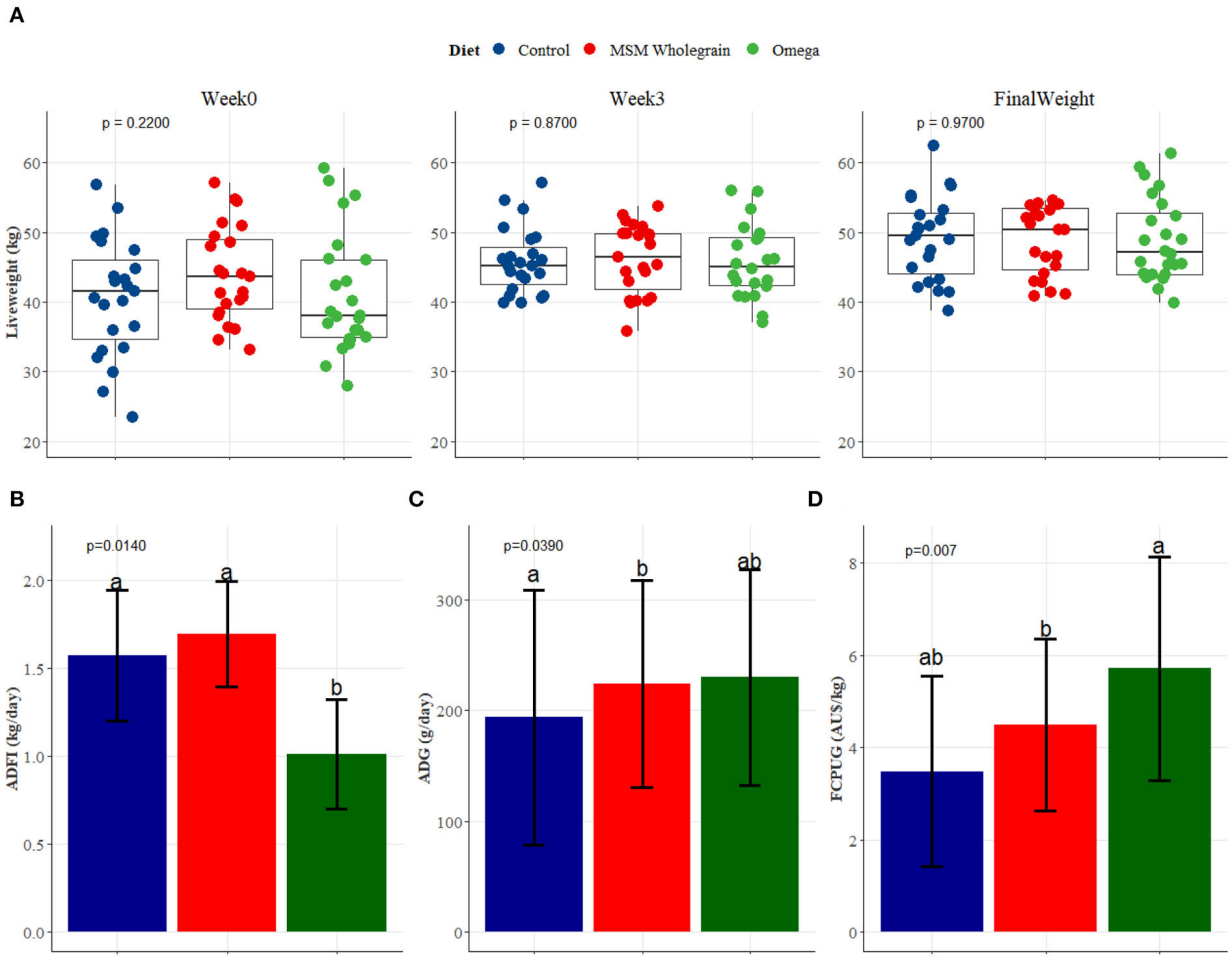
**(A–D)** Liveweights, average daily feed intake, daily gain, and feed cost per unit gain in lot-fed Tattykeel Australian White MARGRA lambs in different treatment groups. Hays without omega-3 (control); whole grain pellets (MSM); hay plus pellets fortified with omega-3 (Omega). ^abc^Superscript letters indicate a significant difference among the means in each variable (Tukey's adjusted).

Figure 1 shows the liveweights, average daily feed intake (ADFI), average daily gain (ADG), and feed cost per unit gain as influenced by treatment. In all treatment groups, the liveweights at commencement and the 3-week adaptation period were similar and not significantly different from each other. Similarly, at the end of the 47-day feeding trial, the final liveweights did not differ significantly between treatments. However, lambs fed the omega-3 diet had a significantly (*p* < 0.0140) lower ADFI (1.01 kg/day) compared to those fed the control (1.57 kg/day) and MSM whole grain (1.69 kg/day) diets. Lambs fed omega-3 diet gained the most weight with an ADG of 230 g/head/day, followed by MSM whole grain (224 g/head/day) and control (194 g/head/day) (*p* < 0.0390). The result also revealed that n-3 LC PUFA-fortified dietary treatment had the lowest cost in terms of ADFI, followed by control, while MSM whole grain was the most expensive feed. The cost of producing a ton of the control diet was Au$381.55, Au$426.44 for MSM whole grain, and Au$528.30 for the omega-3 diets.

### Carcass characteristics, IMF, and FMP

Table 1 shows that IMF and FMP differed significantly among treatments (*p* < 0.0001). The IMF from lambs fed omega-3 pellets was markedly higher than those fed control and MSM whole grains. The FMP of meat from lambs fed omega-3 diet was the lowest at 30.15°C (29.62–31.85°C) than the control at 34.75°C (34.3–35.3°C) and MSM whole grain at 36.8°C (36.8–37.22°C). The diet fortified with omega-3 led to a marked decrease in FMP (*p* ≤ 0.0001). Overall, the median (IQR) pH level of TAW MARGRA lambs was 6.13 (6.06–6.19) and significantly differed between treatments (*p* = 0.0380; Table 1). On the contrary, there was no significant difference in temperature. Using the Kruskal–Wallis test, fortification with omega-3 had no influence on final LWT, GR fat depth, HSCW, and dressing percentage. Regarding cut yields of lot-fed Tattykeel Australian White MARGRA lambs, only wholesale French rack (*p* = 0.0300) and bones (*p* = 0.0190) showed significant differences. Tukey's adjusted tests for pairwise comparisons between treatment groups indicate a significant difference in pH between control and MSM whole grain **(**−0.33, 95% CI: −0.60 to −0.06, *p* = 0.0198). The omega-3 diet had the lowest FMP and highest IMF.

**Table 1:** Median (inter-quartile range) of meat quality and carcass characteristics in lot-fed Tattykeel Australian White MARGRA lambs in different treatment groups.

**Variables**	**Overall**	**Control**	**MSM**	**Omega**	***p*-Value^1^**
**Meat quality**					
pH	6.13 (6.06–6.19)	6.19 (6.16–6.22)^a^	6.08 (6.06–6.13)^b^	6.12 (6.02–6.17)^ab^	0.038
Temperature (°C)	23.23 (22.42–23.86)	22.37 (21.86–23.36)	23.60 (23.2–23.97)	23.29 (22.79–23.98)	0.100
FMP (°C)	34.75 (31.70–36.80)	34.75 (34.3–35.30)^a^	36.80 (36.8–37.22)^b^	30.15 (29.62–31.85)^c^	0.001
IMF (%)	3.50 (2.73–4.07)	3.50 (3.15–3.58)^a^	2.40 (2.02–2.68)^b^	4.15 (4.03–4.42)^c^	0.001
**Carcass characteristics**					
Final LWT (kg)	48.20 (45.03–52.38)	46.95 (43.67–51.67)	51.23(47.94–53.42)	46.52 (45.27–51.47)	0.640
GR fat depth (cm)	16.00 (14.00–18.00)	16.00 (13.25–17.50)	15.50 (14.00–18.25)	15.50 (14.00–18.75)	0.830
Fat score	5.00 (5.00–5.00)	5.00 (5.00–5.00)	5.00 (5.00–5.00)	5.00 (5.00–5.00)	0.790
HSCW (kg)	23.75 (22.72–26.45)	23.35 (22.00–25.52)	25.95(24.15–26.67)	23.25 (22.72–25.45)	0.200
Dressing (%)	50.18 (49.13–52.23)	49.72 (48.77–50.89)	51.17(50.37–53.69)	49.78 (49.00–51.08)	0.098
**Cut yields (kg)**					
Eye of loin	0.41 (0.36–0.46)	0.46 (0.37–0.50)	0.43 (0.36–0.44)	0.37 (0.34–0.43)	0.190
French rack	1.89 (1.63–2.07)	1.90 (1.76–2.04)^a^	2.08 (1.96–2.32)^ab^	1.71 (1.59–1.80)^b^,	0.030
Tenderloin	0.22 (0.20–0.24)	0.22 (0.22–0.24)	0.23 (0.2–0.26)	0.22 (0.18–0.24)	0.690
Banjo shoulder	2.53 (2.28–2.67)	2.55 (2.40–2.79)	2.44 (2.28–2.57)	2.41 (2.26–2.64)	0.380
Neck	0.58 (0.48–0.68)	0.51 (0.47–0.66)	0.62 (0.52–0.72)	0.49 (0.48–0.58)	0.310
Leg shank	5.47 (5.21–5.73)	5.60 (5.47–5.77)	5.37 (5.22–5.52)	5.18 (4.95–5.73)	0.190
Rump	1.05 (0.94–1.15)	1.07 (1.04–1.19)	0.98 (0.94–1.04)	1.01 (0.94–1.04)	0.150
Rib set	0.77 (0.66–0.88)	0.75 (0.70–0.84)	0.80 (0.64–0.94)	0.71 (0.66–0.85)	0.770
Breast and flank	0.88 (0.80–0.96)	0.90 (0.80–1.03)	0.88 (0.79–0.95)	0.88 (0.84–0.94)	0.860
Lean trim	2.96 (2.56–3.40)	3.19 (3.11–3.71)	2.74 (2.58–3.01)	2.65 (2.42–3.17)	0.130
Fat trim	2.27 (1.77–2.77)	2.05 (1.50–2.62)	2.46 (1.82–3.29)	2.10 (1.78–2.62)	0.420
Bones	4.00 (3.46–4.34)	4.30 (4.01–4.64)^a^	4.04 (3.48–4.41)^ab^	3.55 (3.29–3.91)^b^	0.019
EMAW	64.13 (60.00–69.50)	62.00 (60.25–67.50)	65.00 (60.5–72.25)	61.00 (60.00–67.00)	0.510
Total retail meat yield	9.23 (7.94–10.20)	10.13 (9.06–10.21)	9.26 (7.88–10.43)	8.34 (7.91–9.53)	0.220
Trims & bones	7.53 (6.90–8.09)	7.63 (7.32–8.37)	7.50 (6.62–7.95)	7.28 (6.85–8.20)	0.410
Saleable meat yield	16.77 (15.74–18.05)	17.57 (16.04–18.18)	16.12(15.83–17.38)	15.58 (14.79–16.86)	0.230

^1^Based on Kruskal–Wallis test; hays without omega-3 (control); whole grain pellets (MSM); hay plus pellets fortified with omega-3 (omega); fat melting point (FMP); intramuscular fat (IMF); hot standard carcass weight; LWT liveweight (HSCW); eye muscle width (EMAW); GR fat depth; hot standard carcass weight (HSCW); liveweight (LWT).

^abc^Superscript letters indicate a significant difference among the means in each row (Tukey's adjusted).

### Correlations

**Figure 2:**
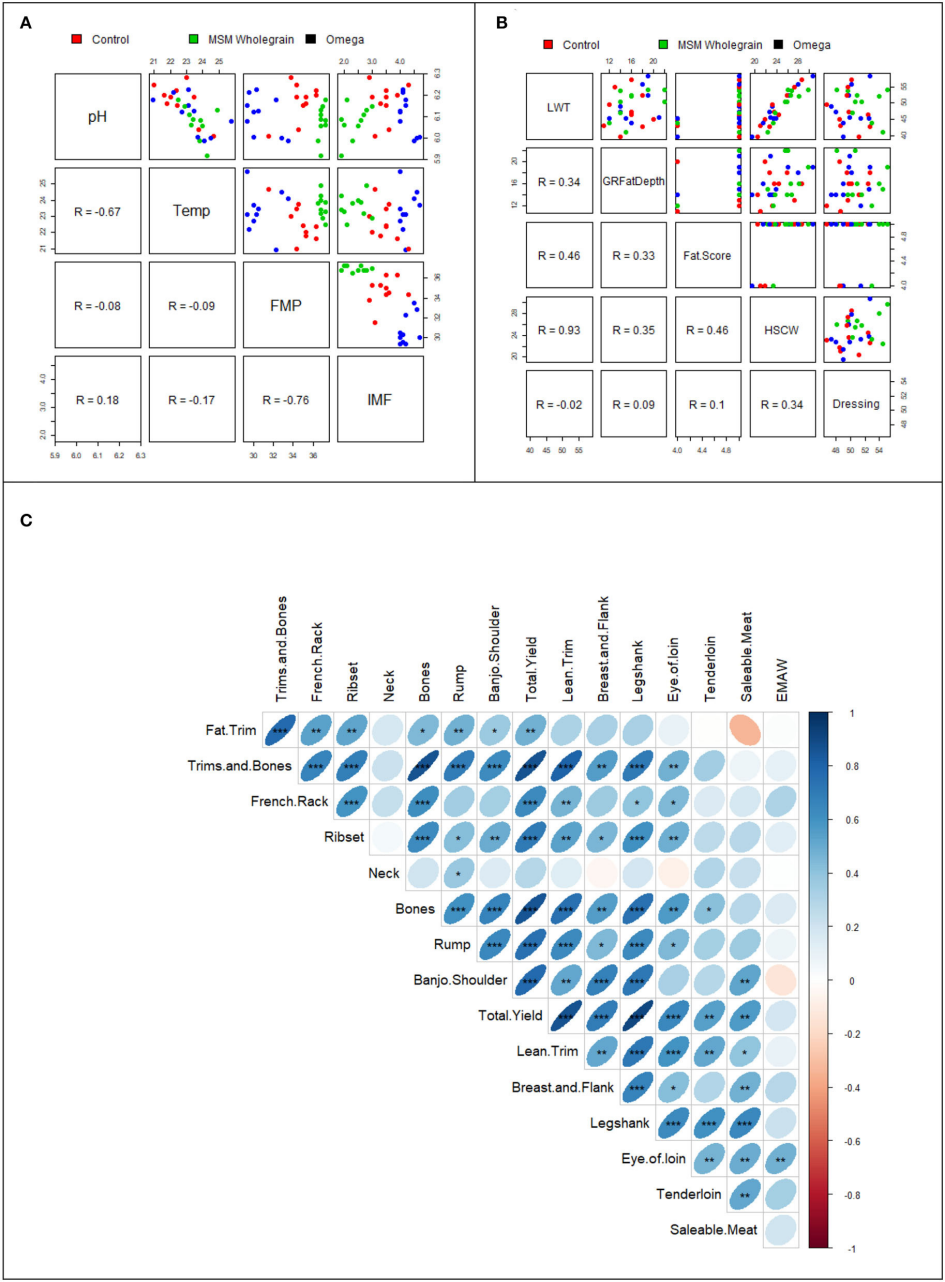
Correlations between **(A)** meat quality indices, **(B)** carcass parameters, and **(C)** cut yields of lot-fed Tattykeel Australian White MARGRA lambs. Hays without omega-3 (control); whole grain pellets (MSM); hay plus pellets fortified with omega-3 (omega); GR fat depth; hot standard carcass weight (HSCW); liveweight (LWT). ***p = 0.001, **p ≤ 0.01, *p ≤ 0.05.

There was a significantly strong negative correlation between temperature and pH (*r* = −0.67, *p* < 0.05) and between FMP and IMF (*r* = −0.76, *p* < 0.05; Figure 2A). As shown in Figure 2B, there was a very strong positive and significant relationship between LWT and HSCW (*r* = 0.93), while a moderate and positive relationship (*r* = 0.46) was observed between LWT and fat score and between fat score and HSCW (*r* = 0.46). Moderately positive and significant correlations (*p* < 0.001) were observed between fat trim and total yield, bones, and eye of loin (*p* < 0.001), lean trim and tenderloin (*p* < 0.001) in Figure 2C. There was a weak and negative correlation between saleable meat and fat trim (*p* < 0.001).

## Discussion

The n-3 LC-PUFA, especially DHA (docosahexaenoic acid; C22:6n-3) and EPA (eicosapentaenoic acid; C20:5n-3), are mainly found in large quantities in oily cold-water fish and seafood, but in insufficient amounts in ruminant meat and milk (32). The n-3 LC-PUFA are known to perform vital physiological roles in maintaining and growing fetuses, neonates, and infant brains (33). The low concentration of n-3 LC PUFA in ruminant muscle tissue is mainly due to extensive lipolysis and biohydrogenation of unsaturated fatty acids (UFA) by ruminal microbes (34, 35). The content of a lamb's diet influences the composition and value of its tissues (36). Furthermore, several trials have indicated that numerous feeding approaches can facilitate the deposition of n-3 LC PUFA in muscle tissues in lambs, resulting in healthier meat (16, 24, 37–42). The use of vegetable seed oils in ruminant diets was found to increase energy and the level of UFA deposited in meat (43). Thus, the resultant meat product had reduced levels of FA that were termed undesirable and enhanced levels that were characterized as beneficial to human health (44). Meat quality indices can be improved by the inclusion of lipid supplements in diets fed to animals raised under feedlot conditions to increase the levels of oleic, linoleic, and linolenic acids and other n-3 LC-PUFA (45, 46). To the best of our knowledge, this is the first study that evaluated the supplementation of diets in TAW MARGRA with promising results.

### Liveweight, average daily feed intake, average daily gain, and feed cost

The inclusion of omega-3 oils decreased dry matter intake but led to significantly higher ADG, an indication of better feed conversion efficiency. The higher average daily gain with less feed consumption implies a better utilization of absorbed nutrients from the abomasum either due to a modification of the rumen environment and ecology that favors less biohydrogenation and more by-pass proteins from the rumen or a higher turnover rate of volatile fatty acid absorption. In economic terms, the relatively higher feed cost of the omega-3 fortified diet was offset by the highest feed efficiency of consuming less but growing the fastest in terms of average daily gains, thus leading to improved profitability (47). Therefore, fortification of diets with omega-3 resulted in better feed conversion efficiency, and this may assist lot-fed lambs to attain finishing weight early, thus saving costs and improving profitability for sheep farmers.

### Intramuscular fat

Fats are essential nutrients associated with vital physiological functions and are widely distributed within animal tissues and organs such as subcutaneous, intermuscular, and intramuscular fat. Malgwi et al. (48) defined IMF as the quantity of fat resident in meat also referred to as marbling fat. In lambs, Pannier et al. (49) reported IMF values of 1.5–9.5%. In this current study, lambs fed omega-3 diet had IMF values between 1.5 and 9.5% (49) recommended for consumers. Australian lamb consumers prefer a 4–5% IMF threshold for palatability and tenderness (50). Lambs fed control diet had IMF values between 3 and 4%, while lambs fed MSM whole grain diets had IMF values between 2 and 3%. IMF has a significant influence on tenderness, flavor, and juiciness, overall liking (51), meat processing (52), and water holding capacity (53). Tenderness, according to Zhao et al. (54), is an essential factor for the valuation of meat quality, and it affects consumer purchasing and market acceptability decisions. IMF is influenced by both genetic and environmental factors, depending on species, breed, genotype, muscle type, age, gender, and nutritional status (55, 56). Consequently, increasing the level of IMF is fundamental to improving meat quality (56, 57).

The n-3 LC-PUFA profile of muscle tissue and organs of TAW MARGRA lambs in this experiment had been published by Pewan et al. (21). Herein, lambs fed the omega-3 fortified diet had better growth performance indicators than the lambs fed the control and MSM whole grain diets.

Generally, consumers prefer lean lamb with low SFA and high n-3 LC-PUFA. The association between total fat content and relative proportions of fatty acids has long been established owing to the minor impact of membrane phospholipids (58, 59). Furthermore, Scollan et al. (57) reported that the potential for IMF accumulation rests on the equilibrium between uptake, synthesis, and degradation of triacylglycerols. This enhances the accessibility of net energy for fat production during finishing and results in higher IMF content.

### Fat melting points

The proportions of single and double bonds influence FMP constituents of fatty acids. For instance, the SFA stearic acid (18:0) and UFA α-linolenic acid (18:3) have melting temperatures of 69.7 and −11°C, respectively (60). The UFA are softer with little heat or energy required to melt them compared to SFA, which are harder requiring more energy to melt. Therefore, the omega-3 fortified diet increased the level of UFA in the muscle tissue, thus accounting for the low FMP recorded in this study.

### Muscle pH

The mean pH values (>6.00) obtained were slightly outside the range of 5.3–5.8 after 24 h of slaughter reported by Yagoubi et al. (61), but there were no incidences of dark, firm, and dry (DFD) muscles. This study's results were in accordance with the report of Inserra et al. (62), who fed lambs on diets containing 0% citrus, 24% citrus, and 35% citrus, but in contrast to those of Chiofalo et al. (35) and Ozdogan et al. (63) in cattle and lambs supplemented with olive oil cake. A series of physiological processes, especially glycolysis, occurs before muscles are converted to meat. Under anaerobic conditions after slaughter, the glycogen stored in muscle tissues is converted to lactic acid, leading to a drop in pH (64) and the onset of rigor mortis. Stenberg et al. (64) reported that lambs fed high-energy diets tend to have higher pH values than their counterparts fed low-energy diets. These lambs could have lost a lot of glycogen during transport from the feedlot facility to the abattoir. Generally, muscle pH is a significant indicator of *post-mortem* animal muscle glycolysis, which is related to water-holding capacity and meat color (65, 66). Lower pH values make the muscle more acidic, bacteriostatic, and fungistatic, thereby hindering bacterial and fungal growth. Lambs fed energy-dense diets have a better capacity to store and replenish glycogen in the muscle tissue and are more capable of coping with pre-slaughter processing, including transport (45). According to Holman et al. (25), the degree of pH decline is significant as meat tenderness is dependent on it. When muscle temperatures decline rapidly, the meat assumes a more rigid state and cold shortening ensues.

### Muscle temperature

Dietary treatment did not influence (*p* > 0.05) temperature, probably due to identical protein, carbohydrate contents, and dry matter intake (45, 67). It is highly unlikely that other physiological factors could have affected the observations in muscle pH since all the animals were wethers of the same breed and age. Stress and excessive exercise before slaughter and electrical inputs during dressing should be minimized as much as possible to reduce muscle temperature.

### Wholesale commercial meat cut yields

Lamb cuts have been designed and marketed based on their nutritional quality, offering processors and retailers the ability to use heavy lambs more efficiently (68). The percentage of bone has been reported to be higher and edible tissues lower in younger than older animals (69). This implies that other tissues develop with advancing age, where the proportion of edible tissues rises as the animal ages. This, unfortunately, has its drawbacks, as the meat becomes less tender, but has a more intense odor and flavor (70). Carcasses of lambs on the omega-3 fortified diet were of better conformation, hence the superior French rack yield, more saleable meat, and lean-to-bone ratio than their counterparts fed both MSM and control diets. Body measurements are vital in estimating liveweights for farm animals (71), and this valuable information contributes to decisions in terms of selection and husbandry system aimed at raising the edible lean meat yield and reduction in fat content in carcasses (5). In sheep and goats, cut yields are predictors of overall carcass tissue composition (72). In this study, the measures of the primal cut weights were identical in the three groups, except for bones and French racks. The proportions of bone, muscle, and fat shift during the growth of an animal concurrently with its carcass water, protein, fat, and mineral contents. These are influenced by the animal's age, weight, breed, sex, nutritional status (73, 74), and production system. The weight and yield of carcasses are significant determinants of commercial value with better returns (75). The mean tissue content of the leg showed that the muscles had the greatest contribution (66.70%), followed by bone (18.89%) and fat (10.09%) (76). Bautista-Díaz et al. (77) reported that bones made up 1.46 ± 0.27 kg of suckling lambs. On account of the weights of primal cuts recorded in this study, the carcasses provided reasonable cut yields when the leg, loin, and shoulder were considered (78). Da Trindade Silva et al. (76) reported that these are responsible for an estimated 60% of the entire yield of cuts. Results generated from this study with other carcass traits could therefore be useful when making decisions regarding the selection and the most appropriate husbandry system to employ.

## Conclusion

The results of this study showed that dietary fortification with n-3 LC-PUFA enhanced feedlot performance in TAW MARGRA lambs with significant improvement in health-beneficial intramuscular fat content, low-fat melting point, and French rack primal cut yield. The results align with the tested hypothesis that the inclusion of n-3 LC-PUFA in feedlot diets will improve productive performance, carcass characteristics, wholesale commercial French rack primal cut yields, and meat quality traits in TAW MARGRA lambs. The inclusion of omega-3 oils in feedlot diets decreased dry matter intake, increased feed efficiency resulting in faster growth, and healthier meat from supplemented lambs.

## Data availability statement

The original contributions presented in the study are included in the article/supplementary material, further inquiries can be directed to the corresponding author.

## Ethics statement

The animal study was reviewed and approved by James Cook University Animal Ethics Committee (Permit No. A0015657).

## Author contributions

Conceptualization, software, project administration, and funding acquisition: AEOM-A. Methodology: AEOM-A, JRO, SBP, OAA, and RTK. Validation: AEOM-A, SBP, and JRO. Formal analysis: SBP and OAA. Investigation: AEOM-A, JRO, SBP, OAA, and RTK. Resources: AEOM-A, RTK, and OAA. Data curation and writing-original draft preparation: SBP. All authors have read and agreed to the published version of the manuscript.

## Funding

This study was funded by the Australian Commonwealth Innovations Connections Research Grant, the Science and Industry Endowment Fund (SIEF) Ross Metcalf STEM Business Industrial Research Fellowship jointly funded by Tattykeel Australian White Pty Ltd (awarded to JRO), and a PhD Research Scholarship from the James Cook University, Townsville, Queensland, Australia (awarded to SBP).

## Conflict of interest

The authors declare that the research was conducted in the absence of any commercial or financial relationships that could be construed as a potential conflict of interest.

## Publisher's note

All claims expressed in this article are solely those of the authors and do not necessarily represent those of their affiliated organizations, or those of the publisher, the editors and the reviewers. Any product that may be evaluated in this article, or claim that may be made by its manufacturer, is not guaranteed or endorsed by the publisher.
